# Building a Sustainable Future: Lessons from the evaluation of the Quality of Care Network for improving maternal, newborn and child health

**DOI:** 10.1371/journal.pgph.0004308

**Published:** 2025-02-21

**Authors:** Blerta Maliqi, Olive Cocoman, Martin Dohlsten, Nuhu Yaqub, Anshu Banerjee

**Affiliations:** 1 Patient Safety and Quality of Care Unit, World Health Organization, Geneva, Switzerland; 2 Maternal Newborn Child Adolescent Health and Ageing Department, World Health Organization, Geneva, Switzerland; 3 UNICEF, Abuja, Nigeria; PLOS: Public Library of Science, UNITED STATES OF AMERICA

## Background

Quality of care (QoC) – meaning care that is effective, safe, people-centred, efficient, integrated, timely and equitable - remains a persistent global challenge that undermines maternal and newborn health (MNH) improvements, particularly in low- and middle-income countries (LMICs) [1]. By 2016 LMICs developed national strategies and action plans for MNH; however, the absence of institutional mechanisms and effective implementation frameworks limited the scale and impact of these efforts.

In response, Bangladesh, Côte d’Ivoire, Ethiopia, Ghana, India, Malawi, Nigeria, Sierra Leone, Uganda, and the United Republic of Tanzania, collaborated with WHO and partners to launch the Network for improving quality of care for maternal, newborn and child health (the Network) in 2017. This initiative emphasized strong national strategic leadership to advance quality health services supported by robust technical multi-stakeholders’ partnerships. The approach prioritized building alliances, generating shared learning to address common challenges, and drawing attention to the need for a health system-enabling environment to sustain and scale-up QoC implementation [2].

## Quality of Care Network (QCN) Evaluations

The QCN Evaluation series provides an in-depth analysis of the effectiveness, legitimacy and sustainability of QoC initiatives in four of the 11 Network countries -Bangladesh, Ethiopia, Uganda and Malawi - during 2017-2022. Consistent with existing Network documentation, the findings highlight significant variation in progress and the unique approaches across countries that shaped the advancement of leadership, action, learning, and accountability for quality MNH [2–6] (Box 1).

Box 1 **Lessons Learned from the QCN Evaluation Series (2017–2022)**

**1. Strong Leadership and robust partnerships.**

Development of QoC policies and management structures by all Network countries demonstrated the importance of commitment and leadership structures. Countries with strong technical partnerships advanced faster. Nevertheless, challenges such as resource constraints, reliance on external partners, and the COVID-19 pandemic disrupted progress. Clarifying roles and responsibilities of each actor remains key.

**2. Action supported by sub-national plans are pivotal.**

Investments at district and facility levels, including mentorship, adaptation of QoC standards, and integration into existing MNH programs, proved essential for advancing QoC implementation.

**3. Collaborative learning driving sustainability and scaling up.**

Facility- and district-based networks strengthened implementation, while South-South collaboration facilitates integration and systematized learning. Institutionalizing learning systems, such as those in Uganda and Malawi, are the basis for continuity and scale.

**4. Data capture and use, and instituting accountability mechanisms is challenging and demonstrating impact takes time**

Participatory mechanisms that facilitate the demonstration of accountability are fundamental to success. However, weak information systems and the need to develop robust performance frameworks for quality of care need to progress more rapidly and become part of routine monitoring and improvement processes.

Firstly, in terms of leadership, Shawar et al findings on differential progress align with existing literature emphasizing the critical influences of pre-existing leadership structures, stakeholder relationships, and resource availability [6]. Over time, all Network countries developed or updated QoC policy and strategies, established management structures and engaged or established multi-stakeholder technical working groups for quality and MNH, with all Network countries achieving this milestone by 2023 [3,4]. However, as documented in the series by Akter [7] (2023) and elsewhere by Cocoman [8], some countries faced challenges due to unclear roles and responsibilities between newly established QoC departments and MNH programs, leading to tensions in leadership, offering lessons for other countries [7,8].

Regarding coalition building, findings from Djellouli et al, Dube et al & Tefsa et al align with existing Network documentation, showing that countries with pre-existing partnerships to support QoC achieved quicker progress. However, many faced challenges, including resource constraints, disruptions caused by the COVID-19 pandemic, and overreliance on external partners for implementation [2–4,7,9–11]. In Ghana and Sierra Leone, sustainability and scaling of QoC initiatives were linked to robust coordination and country led financing from internal and external stakeholders [2,4]. Persistent barriers to progress include insufficient facility level resources, inadequately supported human resources, and inadequate country-led financing, as highlighted by Tesfa [1,11].

Investments by partners at the sub-national level, particularly in districts and facilities, are highlighted in the series as critical to addressing these challenges and advancing QoC implementation [2,6,12]. Network progress tracking also identifies that sub-national adaptation of QoC standards - used to inform training and build capacity in quality improvement (QI) and MNH clinical care proved pivotal in Ethiopia, Ghana, Nigeria, Sierra Leone, and Uganda [2]. Regional and district health management teams maintain momentum through mentorship, data use, learning documentation and the integration of QoC into MNH programming, such as in maternal and perinatal death audits, clinical mentorship, and preservice training ensuring continuity [12].

Collaborative learning and documenting best practices remain critical, as emphasised in multiple papers in the Series [5,7,11,13,14]. Mukinda et al highlighted the importance of facility and district hub networks as a Network strength, though bi-directional knowledge sharing between countries and harmonized methods posed challenges [2,6,12]. This was recognized as a limitation in the four countries studied; nonetheless it is important to acknowledge that learning documentation and sharing requires time to mature, as well as capacity and prioritization at the country level. Learning systems gained momentum as QoC activities progressed, supported by the global secretariat’s focus on South-South collaboration and technical guidance on establishing learning systems at facility, district, and national levels fostering integration between these levels [9,13]. By 2023, countries like Uganda and Bangladesh formalised national learning agendas with QI collaboratives. Not documented are the annual learning cycles, involving broad subnational and national exchanges, held consistently since 2021 in Ethiopia, Ghana, and Sierra Leone. Further, Malawi’s National Learning Centre for QoC is emerging as regional learning hub. This growing institutionalisation of learning systems for QoC is essential to the continuity of progress. Equally, accountability

## Future considerations

Ensuring the continuity of QoC systemic efforts within evolving health systems remains a priority for all countries that are aiming to achieve the SDGs. These countries face significant challenges amidst leadership turnover, fluctuating staffing, lack of quality improvement capabilities, systemic shocks and ongoing changes [3,13]. Continuity of investments is critical to institutionalisation and scaling up QoC-supporting structures and maintaining learning structures and capacities. While some countries have institutionalized QoC learning systems, gaps remain across all Network countries in data collection, integration of QoC indicators into national health information systems (HIS) and their use for quality improvement, decision-making, and impact demonstration [2,15]. Sharing best practices relies on comprehensive documentation and practitioner-generated data, yet only four countries (Ghana, Malawi, Nigeria, and Sierra Leone) have integrated common MNH QoC indicators into their HIS [1,4]. Several factors hinder progress, including delays in setting up QoC monitoring systems, lack of synchronisation between the roll-out of QoC activities and monitoring efforts, and inconsistent facility and district-level data sharing [2,15]. As part of routine monitoring, participatory mechanisms facilitate the demonstration of accountability are fundamental. These were documented in the Series as nascent by 2022, and are emerging in countries (Ghana, Malawi), and require support as institutionalising requires capacity building and time [2,11].

New challenges, such as the developing systems and practices that continuously reward quality -through financial incentives or systems based on quality of care - the implementation of QoC assessment mechanisms that allow a quality improvement pathway to be charted based not on projects but on outcomes of performance improvement; the institutionalisation of patient, institutionalising patient, family and community involvement in shared decision-making in health services that ensure safety for all; and investing in working environments that allow health workers to provide care with dignity and not under pressure and fear of making mistakes, are becoming more urgent and need addressing. Ministries of health and other implementers continue to need technical support to chart these challenges.

Looking ahead, consistently prioritising quality at all levels of the health system and ensuring its continuity is essential to sustain and build on progress achieved. Countries need to consolidate QoC leading and implementing structures, establish stronger mechanisms for data collection, utilisation, best practice documentation, and policy dialogues with decision-makers and communities. National academic and research institutions can play a critical role in strengthening these processes supported by regional or global systems that foster South-South learning. Aligning partners’ technical and financial investments will reinforce national leadership, sustain progress, and scale quality initiatives. The Network has established vital groundwork for improving the quality of MNH care and the whole health system, however, sustained focus on system adaptability, resource alignment, and learning will be essential for continuity and future advancements.
